# Genetic algorithm learning in a New Keynesian macroeconomic setup

**DOI:** 10.1007/s00191-017-0511-y

**Published:** 2017-07-04

**Authors:** Cars Hommes, Tomasz Makarewicz, Domenico Massaro, Tom Smits

**Affiliations:** 1 CeNDEF, University of Amsterdam, Amsterdam, Netherlands; 2 Tinbergen Institute, Amsterdam, Netherlands; 3 Complexity Lab in Economics, Universitá Cattolica del Sacro Cuore, Milan, Italy; 4 SEO Amsterdam Economics, Amsterdam, Netherlands

**Keywords:** Expectation formation, Learning to forecast experiment, Genetic algorithm model of individual learning, C53, C61, C63, C92, E12, E31, E52

## Abstract

In order to understand heterogeneous behavior amongst agents, empirical data from Learning-to-Forecast (LtF) experiments can be used to construct learning models. This paper follows up on Assenza et al. (2013) by using a Genetic Algorithms (GA) model to replicate the results from their LtF experiment. In this GA model, individuals optimize an adaptive, a trend following and an anchor coefficient in a population of general prediction heuristics. We replicate experimental treatments in a New-Keynesian environment with increasing complexity and use Monte Carlo simulations to investigate how well the model explains the experimental data. We find that the evolutionary learning model is able to replicate the three different types of behavior, i.e. convergence to steady state, stable oscillations and dampened oscillations in the treatments using one GA model. Heterogeneous behavior can thus be explained by an adaptive, anchor and trend extrapolating component and the GA model can be used to explain heterogeneous behavior in LtF experiments with different types of complexity.

## Introduction

In this paper, we study a simple New Keynesian economy, in which the individuals optimize a forecasting heuristic with a genetic algorithms optimization procedure. We show that this GA-model, taken almost directly from Anufriev et al. (2015), is able to replicate well the main findings of an experimental study by Assenza et al. (2013). The GA learning model therefore explains individual (micro) as well as aggregate (macro) behavior of different laboratory economies.

In dynamic macroeconomic models, such as the standard New Keynesian model, expectation feedback plays an important role in the shape and stability of economic equilibria. Traditional literature (Muth 1961) would disregard the potential heterogeneity of forecasting behavior and focus instead on the model consistent Rational Expectations. However, limitations on individual rationality are likely to prevail (e.g. Cornea *et al*. 2017), especially when the individuals need time to learn the structure of the economy (Sargent 1993). Once we allow for bounded rationality, a non-linear price expectations feedback can lead to complicated and potentially volatile dynamics and multiple equilibria (Anufriev et al. 2013a). On the other hand, there are many different forecasting rules that individuals can use to form their expectations about future prices. It is, therefore, important to study how such rules are selected in a realistic learning environment, and how learning in the context of macroeconomics relates to forecasting in other economic settings, including financial or commodity markets.

In order to study the individual forecasting behavior in a controlled laboratory environment, Learning-to-Forecast (LtF) experiments have been introduced (Marimon et al. 1993). The role of human subjects is to forecast prices, which are then translated into realized prices through some market mechanism, such as a simple supply-driven cobweb economy in which the subjects are framed as advisers to the commodity producers. The LtF experiments typically have a straightforward and unique fundamental equilibrium, and hence can be directly used to assess individual learning dynamics. In practice, they show that individuals indeed have heterogeneous expectations (Heemeijer et al. 2009; Hommes 2011; Anufriev and Hommes 2012), which greatly depend on the specific structure of the feedback market. Moreover, subjects can coordinate away from the fundamental equilibrium, or even on oscillatory time paths (Hommes et al. 2005; Assenza et al. 2013).

In order to understand this heterogeneous behavior, the LtF experimental data can be used to construct and assess learning models. A notable example is work by Anufriev and Hommes (2012), who adapted the Brock-Hommes model (Brock and Hommes 1997) into a Heuristic Switching Model (HSM) with four simple rules, and apply it to explain the experiment of Hommes et al. (2005). Assenza et al. (2013) use the same model to explain their experimental findings in a New Keynesian setting, while Anufriev et al. (2013b) fit a 2-type HSM to explain the difference between aggregate behavior in positive versus negative feedback systems. In general, HSM remains a versatile model that can approximate the individual learning of forecasting behavior across different experiments.

A generalized, agent-based counterpart of HSM is the model of individual learning based on genetic algorithms (GA; Haupt and Haupt, 2004). GA is an optimization method based on a population of arguments that compete on their function value and can therefore be applied to a wide class of problems: they rely on an intelligent search of a large but finite solution space using statistical methods and can deal with discrete variables and noncontinuous cost functions (Haupt and Haupt 2004). Arifovic (1991) has developed an augmented GA model, which was consequently applied in different economic settings such as a cobweb model (Arifovic 1994; Hommes and Lux 2013) and an overlapping generations model (Arifovic 1995). Following a more developed version of the model by Hommes and Lux (2013), Anufriev et al. (2015) have shown that a model in which individuals independently optimize their prediction rules using GA is able to replicate experimental findings from three different LtF experiments, based on commodity or financial markets. A great advantage of this approach is that this model is a generalized version of the HSM without pre-specification of forecasting rules, and hence can be used to motivate the parametrization of simple HSM’s (Anufriev et al. 2015).

This paper follows up on Assenza et al. (2013) by using a GA model to replicate the results from their LtF experiment based on a New Keynesian macro model. We use the same GA model as Anufriev et al. (2015), i.e. we update heuristics with an adaptive, an anchor an a trend extrapolation coefficient. In this way, we contribute to understanding LtF experiments in a New Keynesian environment using GA. Unlike Arifovic et al. (2012) who have investigated GA in a New Keynesian environment as well, we explain the heterogeneous behavior with heuristics that depend only on the realizations and previous predictions by the agent, similar to the heuristics used by Heemeijer et al. (2009), Anufriev and Hommes (2012) and Assenza et al. (2013). Moreover, we use experimental settings with different types of complexity and show that LtF experiments with increasing complexities can be explained using the same GA model. This shows that the GA model is versatile and can replicate varied experimental economies, but also remains robust against the *object of the forecasting task*. Our paper thus shows that the original GA model by Anufriev et al. (2015) explains the individual learning to forecast not only in a univariate model of prices in a specific market, but also a more complex macrosystem with two variables with expectations feedback and two different groups of forecasters for inflation and output gap.

We replicate results for six different experimental treatments: three different treatments with increasing complexity (1, 2 and 3), each subdivided into two treatments (a and b) with more or less aggressive monetary policy. The results from the treatments 1, 2 and 3 in the experiment can be classified in three types of aggregate behavior, respectively: converging, oscillatory and dampened oscillatory behavior. The main goal of this paper is to show that all three types of behavior can be reproduced using 50-period ahead simulations of one GA model. We use Monte Carlo simulations as in Anufriev et al. (2015) to investigate how well the model explains the experimental data.

The paper is organized as follows. Section 2 describes our model. We give a description of the New Keynesian framework, the experimental treatments and results, and elaborate on the GA model that we use to replicate the experiments. The results are described in Section 3, where we graphically show the replications of the experiment and compare the experiment to our replications using descriptive statistics. In Section 4, we present our conclusions and recommendations.

## Model

During the past decade the New Keynesian (NK) monetary model has been a widely used framework for the analysis of monetary policy, in which inflation expectations play an important role. Branch and McGough (2009) and Massaro (2013) have incorporated bounded rationality at the individual agent level and heterogeneous expectations in the NK model. Assenza et al. (2013) use this model to set up a laboratory experiment.^1^ In order to study the individual expectations process, subjects are asked to forecast the inflation rate under three different scenarios. This section describes the NK model, the experimental setup and an explanation of the experimental results, followed by a description of the GA model.

### New Keynesian model

The New Keynesian model with heterogeneous expectations developed by Branch and McGough (2009) is described by the following equations:

1 $$\begin{array}{@{}rcl@{}} y_{t}\:\:&=&\:\overline{y}^{e}_{t+1}-\varphi(i_{t}-\overline{\pi}^{e}_{t+1})+g_{t} \end{array} $$

2 $$\begin{array}{@{}rcl@{}} \pi_{t}&=&\:\lambda y_{t} + \rho\overline{\pi}^{e}_{t+1}+u_{t} \end{array} $$

3 $$\begin{array}{@{}rcl@{}} i_{t}&=&\:\overline{\pi}+\phi_{\pi}(\pi_{t}-\overline{\pi}) \end{array} $$

In this system, Eq. 1 describes the aggregate demand in which the output gap *y*_*t*_ depends on the average expected output gap $\overline {y}^{e}_{t+1}$ and on the real interest rate $i_{t}-\overline {\pi }^{e}_{t+1}$. Equation 2 shows how the inflation rate depends on the output gap and on average expected inflation. Finally, Eq. 3 is the monetary policy rule implemented by the monetary authority in order to keep inflation at its target value $\overline {\pi }$. In Eqs. 1 and 2, *g*_*t*_ and *u*_*t*_ are small normally distributed errors.^2^

The NK model requires agents to forecast both inflation and the output gap. These forecasts are *2-period ahead* forecasts, since, at the time the forecasts $\pi ^{e}_{t+1}$ and $y^{e}_{t+1}$ are formed, the most recent observations are *π*_*t*−1_ and *y*_*t*−1_. Given that forecasting two variables simultaneously might be a too difficult task for subjects, the experiment in Assenza et al. (2013) has been run using three different treatments.

### Treatments

In the first treatment of the experiment, where only the inflation rate needs to be forecast, the model reduces to a framework with a structure similar to the experimental framework that was used by Anufriev et al. (2015). In this treatment, the expectations on the output gap are fixed at the equilibrium value. In the second treatment, subjects only forecast the inflation rate, and expectations on the output gap are represented by naive expectations, i.e. the last observation. This results in a two-dimensional structure with output-inflation dynamics that makes the macro framework more complicated. The third treatment of the experiment represents an economy driven by individual expectations on two different aggregate variables, with two different groups of forecasters, predicting, respectively, inflation and the output gap.

Moreover, all treatments are run under different monetary policy regimes, a regime *a* in which *ϕ*_*π*_ = 1 and a regime *b* in which *ϕ*_*π*_ = 1.5, where *ϕ*_*π*_ is a policy parameter measuring how strongly the interest rate responds to deviations of inflation from its target.

#### Treatment 1

In the first treatment, subjects forecast inflation, while the expectations on the output gap are assumed to be given by the equilibrium predictor $\:\overline {y}^{e}_{t+1}=(1-\rho )\overline {\pi }\lambda ^{-1}$. The initial set of equations can now be written as:

4 $$\begin{array}{@{}rcl@{}} y_{t}\:\:&=&(1-\rho)\overline{\pi}\lambda^{-1}-\varphi(i_{t}-\overline{\pi}^{e}_{t+1})+g_{t} \end{array} $$

5 $$\begin{array}{@{}rcl@{}} \pi_{t}&=&\:\lambda y_{t} + \rho\overline{\pi}^{e}_{t+1}+u_{t} \end{array} $$

6 $$\begin{array}{@{}rcl@{}} i_{t}&=&\:\overline{\pi}+\phi_{\pi}(\pi_{t}-\overline{\pi}) \end{array} $$

in which $\overline {\pi }^{e}_{t+1}$ is the average prediction of the subjects in the experiment. Substituting (6) into (4) results in:

7 $$\begin{array}{@{}rcl@{}} y_{t}\:\:&=&(1-\rho)\overline{\pi}\lambda^{-1}+\varphi\overline{\pi}(\phi_{\pi}-1)-\varphi\phi_{\pi}\pi_{t}+\varphi\overline{\pi}^{e}_{t+1}+g_{t} \end{array} $$

8 $$\begin{array}{@{}rcl@{}} \pi_{t}&=&\:\lambda y_{t} + \rho\overline{\pi}^{e}_{t+1}+u_{t} \end{array} $$

Solving this in terms of inflation *π*_*t*_ gives:

9 $$\begin{array}{@{}rcl@{}} \pi_{t}&= \frac{(1-\rho)-\lambda\varphi(\phi_{\pi}-1)}{1+\lambda\varphi\phi_{\pi}}\overline{\pi} + \frac{\lambda\varphi+\rho}{1+\lambda\varphi\phi_{\pi}}\overline{\pi}^{e}_{t+1}+\frac{\lambda g_{t}+u_{t}}{1+\lambda\varphi\phi_{\pi}} \end{array} $$

Note that this is a linear relation between *π*_*t*_ and $\overline {\pi }^{e}_{t+1}$, plus a small composite shock as a third term.

#### Treatment 2

In the second treatment, subjects also forecast inflation, but now the expectations on the output gap are assumed to be represented by naive expectations: $\:\overline {y}^{e}_{t+1}=y_{t-1}$. In this case, inflation and the output gap at time *t* can be written as follows:

10 $$\begin{array}{@{}rcl@{}} y_{t}\:\:&=&\varphi\overline{\pi}(\phi_{\pi}-1)-\varphi\phi_{\pi}\pi_{t}+\varphi\overline{\pi}^{e}_{t+1}+y_{t-1}+g_{t} \end{array} $$

11 $$\begin{array}{@{}rcl@{}} \pi_{t}&=&\:\lambda y_{t} + \rho\overline{\pi}^{e}_{t+1}+u_{t} \end{array} $$

in which $\overline {\pi }^{e}_{t+1}$ is the average prediction of the subjects in the experiment. In matrix form, this system of equations becomes:

12 $$\begin{array}{@{}rcl@{}} \left[\begin{array}{lll} \!y_{t}\! \\ \!\pi_{t}\! \end{array}\right] \,=\, \frac{1}{1\,+\,\lambda\varphi\phi_{\pi}} \!\left( \!\!\!\!\!\!\!\phantom{\frac{\frac{1}{1}}{\frac{1}{1}}} \left[\begin{array}{lll} \!0 &\varphi(1\,-\,\phi_{\pi}\rho)\!\!\!\\ \!0& \lambda\varphi\,+\,\rho\! \end{array}\right] \!\! \left[\begin{array}{lll} \overline{y}^{e}_{t\,+\,1} \\ \overline{\pi}^{e}_{t\,+\,1} \end{array}\right] \,+\, \left[\begin{array}{lll} \!\!1 & \!0\\ \!\!\lambda & \!0 \end{array}\!\!\right] \!\! \left[\begin{array}{lll} \!\!y_{t-1}\!\! \\ \!\!\pi_{t-1}\!\! \end{array}\right] \,+\, \left[\begin{array}{ll} \!\!1 & \!-\varphi\phi_{\pi}\!\!\!\\ \!\!\lambda & \!1 \end{array}\right] \!\! \left[\begin{array}{lll} \!g_{t}\! \\ \!u_{t}\! \end{array}\right]\! \right) \end{array} $$

This treatment is more complicated than the first since the inflation does not only depend on expected inflation but also on the lagged output gap as $\overline {y}^{e}_{t+1}=y_{t-1}$.

#### Treatment 3

In the third treatment, two groups participate in the same experimental economy, where one group forecasts inflation and the other group forecasts the output gap. Substituting (3) into (1) and writing the equations in matrix form results in the following system:

13 $$\begin{array}{@{}rcl@{}} \left[\begin{array}{lll} y_{t} \\ \pi_{t} \end{array}\right] =\frac{1}{1+\lambda\varphi\phi_{\pi}} \left( \!\!\!\!\!\!\!\phantom{\frac{\frac{1}{1}}{\frac{1}{1}}} \left[\begin{array}{lll} 1 &\varphi(1-\phi_{\pi}\rho)\\ \lambda& \lambda\varphi+\rho \end{array}\right] \!\! \left[\begin{array}{lll} \overline{y}^{e}_{t+1} \\ \overline{\pi}^{e}_{t+1} \end{array}\right] +\left[\begin{array}{lll} 1 & -\varphi\phi_{\pi}\\ \lambda & 1 \end{array}\right] \!\! \left[\begin{array}{lll} g_{t} \\ u_{t} \end{array}\right] \right) \end{array} $$

In contrast to the first two treatments, treatment 3 represents an experimental economy the aggregate behavior of which is driven by individual expectations on two different interacting aggregate variables.

#### Monetary policy regimes

Each of the three treatments was run under two different monetary policy regimes. In sessions *a*, the coefficient *ϕ*_*π*_ = 1, so that Eq. 3 reduces to *i*_*t*_ = *π*_*t*_. With this setting, there is no attraction whatsoever to target inflation value $\overline {\pi }$. In session *b*, coefficient *ϕ*_*π*_ is set to 1.5 so that the monetary policy responds to inflation aggressively. In this scenario, $\overline {\pi }$ does not drop out of Eq. 3, so that the inflation rate has a tendency towards target value $\overline {\pi }$.

### Experimental results

**Fig. 1:**
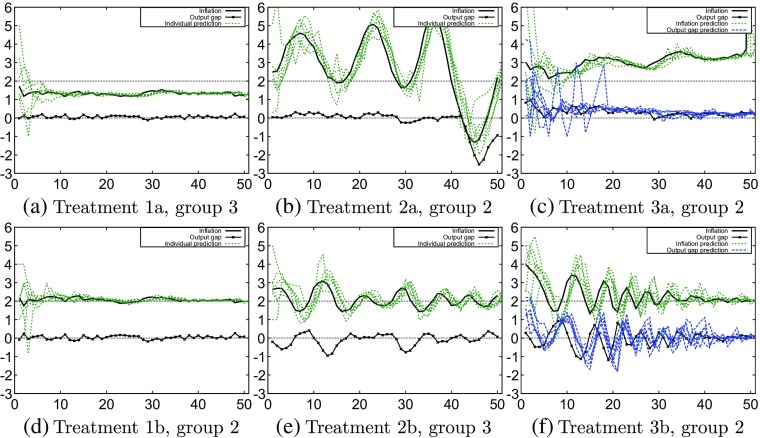
Typical experimental results

The experimental results can be classified into three different types of behavior. Six experimental groups that clearly show these different types of behavior are presented in Fig. 1a through f.

#### **Convergence**

In treatment 1a, two groups converge to a non-fundamental steady state equilibrium (see Fig. 1a). Because the monetary policy responds weakly to inflation rate fluctuations, subjects coordinate on inflation rates other than the target inflation rate. In treatment 1b, two out of three group also converge to a steady state. In this case, however, the monetary policy responds aggressively to inflation, so that subjects tend to coordinate on the target inflation (Fig. 1d).

#### **Oscillations**

In the second treatment, a different type of aggregate behavior can be observed. Subjects in group 2 of treatment 2a converge to an oscillatory pattern (see Fig. 1b). The oscillations are principally above the target inflation rate, due to the lack of an aggressive monetary policy. In treatment 2b, subjects are again forced towards the target inflation rate. The experiment shows small oscillations around the fundamental inflation rate (Fig. 1e).

#### **Dampened oscillations**

The third type of aggregate behavior that this research aims to reproduce is an oscillatory convergence towards a steady state. This behavior occurs in the second session of treatment 3b (Fig. 1f), which starts out with oscillations around the target inflation rate. The oscillations slowly dampen such that there is convergence to the fundamental steady state (which is in line with the monetary policy settings of the b-treatment) near the end of the session.

### The genetic algorithm model

We follow Arifovic’s augmented GA model, in which every individual starts with a set of forecasting heuristics for either inflation rate or the output gap, which are encoded in binary string. After initialization of the model, the heuristics undergo a GA iteration. This iteration, an optimization procedure that uses four evolutionary operators, is the core of the model.

In the GA model, each individual possesses a population of 20 forecasting heuristics, in which one or more parameters need to be optimized. Every heuristic, therefore, entails a candidate vector of optimization parameters encoded in a binary string. This binary string can be seen as a chromosome containing one or more genes - the parameters in the vector. Parameter $\theta ^{n}_{h,i,t}$ is the *n* ^*th*^ parameter in heuristic *h* of individual *i* in period *t*, and is coded in a binary string of length *l* with binary values $g^{n,k}_{h,i,t}$ at the *k* ^*th*^ position in the string as follows:

14 $$ \theta^{n}_{h,i,t}=a_{n}+\frac{b_{n}-a_{n}}{2^{l-1}}\sum\limits_{k=1}^{l}{g^{n,k}_{h,i,t}}2^{k}-1 $$

Since each gene has a finite length, the parameter values are limited to a finite interval, with *a*_*n*_ and *b*_*n*_ as lower and upper boundary, respectively, and to a finite number of different values. The size of this interval, together with the length of the string, determines the precision of the parameter.^3^

#### GA iteration

The encoded heuristic goes through four stages of updating: reproduction, mutation, crossover and election. The operators in the GA iteration are inspired by the theory of evolution, but also have an economic intuition.

The first operator in every GA iteration is the reproduction operator, which randomly draws 20 heuristics for each individual. Every draw takes place according to the heuristics’ probabilities to be chosen for reproduction, based on their performance measure. The reproduction operator represents the phenomenon that more successful strategies (in terms of utility) are more likely to be used in the future.

After reproduction there is a small probability that a *mutation* will occur in the new strategy. In the binary string, each position has an equally small chance of changing from a 0 to a 1 or vice versa. Depending on the position of the string in which the mutation takes place, the effect of a single mutation can be significant or very small.

Combining two different strategies into new strategies is captured by the crossover operator, whereas the mutation operator models small changes in strategies. All 20 heuristics that are picked in the reproduction stage are, after mutation, signed up as random pairs and will interchange a part of the binary string that represents the forecasting coefficients.^4^

In the crossover and mutation stage, two new heuristics are formed from the two old heuristics for the new period. Because these two new heuristics do not always perform better than its predecessors, an election operator tests the performance of the two new and the two old heuristics. The performance of these strings will be based on the difference of the inflation (or output gap) prediction with respect to the last observed inflation (output gap). Out of these four strings, the best performing two will be chosen for the next period.

**Fig. 2:**
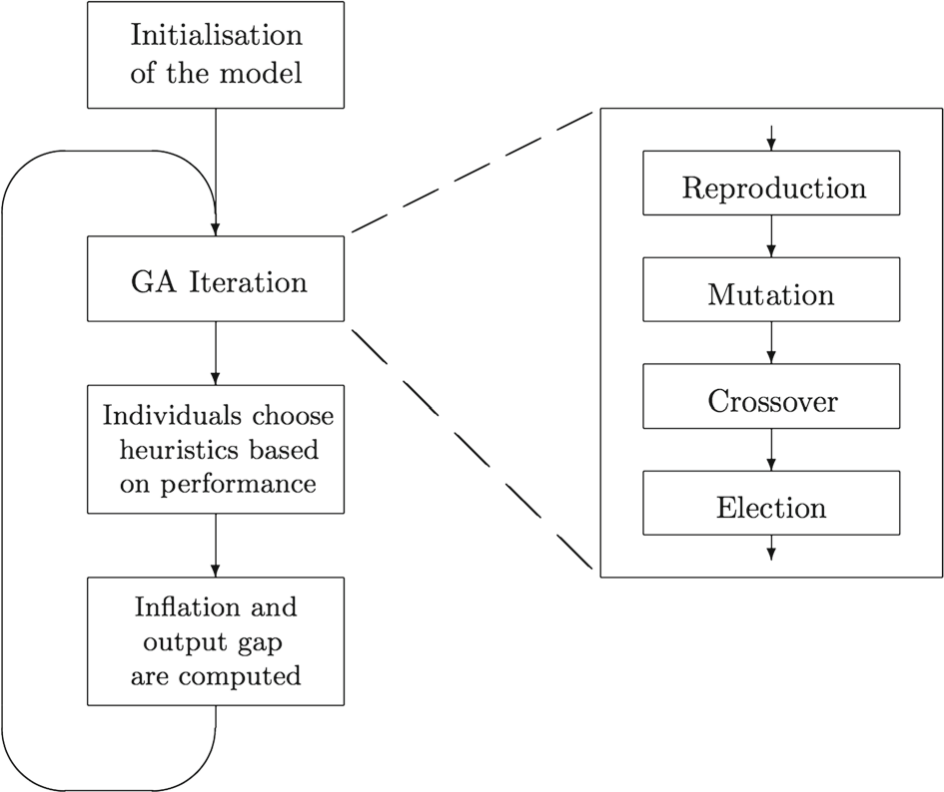
Schematisation of the GA model

After the GA iteration, every individual has an updated set of heuristics at his disposal. From this new set of heuristics, every individual chooses one as his inflation (or output gap) forecast. In order to make this choice, individuals make use of a performance measure for every heuristic: heuristics that perform better, according to this measure, have a higher probability of being chosen. The forecasts of all individuals together determine the first actual value for inflation or output gap according to Eqs. 1, 2 and 3. Individuals now take their updated set of heuristics to the next period, in which the next GA iteration starts. The complete iterative process in the model is illustrated in Fig. 2. This process continues for a number of periods, in which individuals should be able to make better predictions as time goes by, because their heuristics update every iteration.

#### Forecasting heuristics

GA can be interpreted as a generalized version of the forecasting heuristics in the heuristic switching model used by Assenza et al. (2013). Agents do not choose from a set of predefined rules, but use Genetic Algorithms to optimize over a set of parameters in a simple class of linear prediction rules, based on the past inflation and/or output gap, individual past prediction, the observed trend and the average of past inflation and/or output gap.

This general rule consists of an adaptive component (*α*), a trend extrapolating component (*β*) and an anchor component (*γ*). The linear forecast for period *t* + 1 is given by

15 $$ x^{e}_{i,t+1}=\gamma x^{av}_{t-1}+(1-\gamma) (\alpha x_{t-1}+(1-\alpha)x^{e}_{i,t}) +\beta(x_{t-1}-x_{t-2}), $$

where *x*_*t*−1_ is the last observation, *x*_*t*−1_ − *x*_*t*−2_ the last observed change (or trend), $x^{e}_{i,t}$ the last forecast by subject *i* and $x^{av}_{t-1}$ the observed sample average. This rule is in line with the so-called ‘first order heuristic’, which is used by Heemeijer et al. (2009) to explain the participants’ behavior in an experimental economy, and is also used in the GA model by Anufriev et al. (2015). A condition of the first order heuristic is that the coefficients for the anchor [*γ*], the last observed value [(1 − *γ*)*α*] and the last forecast [(1 − *γ*)(1 − *α*)] are non-negative and sum to one. This particular way of formulating the forecasting heuristic ensures this for all values of *α* and *γ* between 0 and 1. Individuals optimize the three coefficients *α*, *β* and *γ*, encoded in a 60-bit string (3 × 20 bits).

The forecasting heuristics described above are used for simulations of all three different treatments. In treatments 1 and 2, only inflation *π* is predicted by the participants, so that the forecasting heuristic simply becomes:

16 $$ \pi^{e}_{i,t+1}=\gamma_{i,h,t}\pi^{av}_{t-1}+(1\,-\,\gamma_{i,h,t})(\alpha_{i,h,t} \pi_{t-1}+(1\,-\,\alpha_{i,h,t})\pi^{e}_{i,t} )+\beta_{i,h,t}(\pi_{t-1\!}-\!\pi_{t-2}) $$

where *α*_*i*,*h*,*t*_, *β*_*i*,*h*,*t*_ and *γ*_*i*,*h*,*t*_ are the parameters of subject *i*, for rule *h*, in period *t*. In treatment 3, however, there are six participants who predict inflation while six other participants predict the output gap. Both variables are updated using the same general rules. Superscripts *π* and *y* are added to coefficients *α*, *β* and *γ* to differentiate between inflation and the output gap:

17 $$\begin{array}{@{}rcl@{}} && {} \pi^{e}_{i,t+1} \,=\, \gamma^{\pi}_{i,h,t}\pi^{av}_{t-1}\,+\,({\kern-.5pt}1\!\,-\,\!\gamma^{\pi}_{i,h,t}{\kern-.5pt})({\kern-.5pt}\alpha^{\pi}_{i,h,t} \pi_{t-1} \,+\, ({\kern-.5pt}1\,-\,\alpha^{\pi}_{i,h,t}{\kern-.5pt})\pi^{e}_{i,t}{\kern-.5pt}) \,+\, \beta^{\pi}_{i,h,t}(\pi_{t\,-\,1}\,-\,\pi_{t-2}) \end{array} $$

18 $$\begin{array}{@{}rcl@{}} && y^{e}_{i,t+1} \,=\, \gamma^{y}_{i,h,t} y^{av}_{t-1}\,+\,(1\!\,-\,\!\gamma^{y}_{i,h,t})(\alpha^{y}_{i,h,t} y_{t-1} \,+\, (1\,-\,\alpha^{y}_{i,h,t})y^{e}_{i,t}) \,+\, \beta^{y}_{i,h,t}(y_{t\,-\,1}\,-\,y_{t-2}). \end{array} $$

#### Performance measure

In this GA framework, every individual has a whole range of forecasting heuristics at hand to forecast inflation (or the output gap) in every period. This choice is made on the basis of the performance of the heuristics, determined by a fitness measure. Hence, the type of performance measure used in the model is of key importance to the simulation process. In the GA model, this performance measure is assumed to be equal to the payoff function used by Assenza et al. (2013) in their experiment, namely:

19 $$ U_{i,h,t}=\frac{100}{1+|\!|x^{e}_{i,h,t-1}-x_{t-1}|\!|}. $$

The performance measure of each heuristic is used twice in every GA iteration: to choose heuristics for reproduction, and to pick one heuristic as a forecast for the next period. In both cases, the probability that a heuristic is chosen is obtained by formalizing the logit-transformation of the utility measure and adding an intensity of choice parameter *β*_*s*_.^5^ This parameter measures the sensitivity of individuals to differences in the performance of their heuristics. This is in line with the performance measure that was used in the HSM by Assenza et al. (2013). The probability that a heuristic is chosen then becomes:

20 $$ {\Pi}_{i,h,t}=\frac{\exp(\beta_{s} U_{i,h,t-1})}{{\sum}^{H}_{h=1} \exp({\beta_{s} U_{i,h,t-1})}}. $$

For all simulations in this research this normalized logit-transformation is used. We choose *β*_*s*_ = 1 in all simulations. The performance measure then simply becomes:

21 $$ {\Pi}_{i,h,t}=\frac{\exp(U_{i,h,t-1})}{{\sum}^{H}_{h=1} \exp({U_{i,h,t-1})}}. $$

### Parametrization

Besides the GA operators, the forecasting heuristics and the performance measure, the model requires the tuning of a few important model settings to replicate the experimental economies. These aspects are discussed in this section.

#### Initialization of the model

Each session in the LtF experiment consists of 50 periods, which means that the GA model should run for the same amount of time. Almost all GA-simulations below will be 50-period ahead simulations. This means that no information from the experiment is used except for the initial predictions by the subjects.^6^ There are two aspects of the initialization of the GA-model: (1) what are the first two predictions in the first period (when there are no past observations yet, and so the forecasting heuristics cannot be used)?, and (2) in the second period (when the heuristics can be used), which coefficients *α*, *β*, and *γ* do the GA agents use? The initialization is done as follows: (1) the initial predictions for periods 1 and 2 are taken from the experimental data (the two initial predictions of each of the six subjects), and (2) the initial parameters *α*, *β*, and *γ* of the forecasting heuristics are randomly chosen (from uniform distributions of the bits in the chromosomes). After this initialization, the model is run for 49 more periods, following the scheme of the GA shown in Fig. 2.^7^

#### Parameters GA model

As explained earlier, the parameters *α*, *β* and *γ* are restricted to a finite interval. For *α* and *γ* the ranges are obvious: the conditions of the first order rule dictate that both *α* and *γ* should be between 0 and 1. The ranges for trend extrapolation parameter *β* are, however, not subject to any constraints. Furthermore, trend extrapolation parameters can, in theory, be negative as well, indicating ‘contrarian’ behavior. Massaro (2012) showed that subjects in the experiment indeed make use of trend extrapolation, but that virtually all subjects use positive coefficients. The range of *β* is therefore set to [0,3] to allow for relatively weak (0 to 1) and relatively strong (1 to 3) trend extrapolation.

Furthermore, the mutation and crossover operators are both subject to a certain probability of occurrence. Throughout the simulations in this research, the mutation rate is set to 0.01, so that during every GA iteration, every bit in every string has a 1% chance of mutating. Crossover does not always happen, either; the crossover rate is set to 0.9, which means that each pair of strings has a 90*%* chance of interchanging a part of the string.^8^

#### Monte Carlo simulations

In order to investigate how well the model explains the experimental data, Monte Carlo simulations are carried out. For all six treatments, simulations of 1000 replications are run. In each run, we draw all initial heuristics (six agents with 20 heuristics each) with parameters *α*, *β* and *γ* drawn randomly from a uniform distribution. The initial conditions for the predicted inflation (output gap) by agents, $\pi ^{e}_{i,1}\: (y^{e}_{i,1})$ and $\pi ^{e}_{i,2}\: (y^{e}_{i,2})$, are the same in every run and equal to the first two predictions by the participants in the corresponding experiment. Shock terms *g*_*t*_ and *u*_*t*_ (see Eqs. 1 and 2) are equal to the shocks used in the experiment and therefore also the same in each run of the Monte Carlo simulations.

These Monte Carlo simulations of 1000 runs of the GA-model enable the computation of confidence intervals. We will compare the experimental results to the mean and the 90% and 95% confidence intervals of the 50-period ahead Monte Carlo GA simulations. Moreover, a confidence interval for some descriptive statistics will be computed. For each treatment, this enables us to compare the mean and standard deviation of 1000 runs of the GA-model to the experimental result.

## Results

This section compares the experimental results to the mean and the 90% and 95% confidence intervals of the Monte Carlo simulations, illustrated graphically. Additionally, confidence intervals of the mean and standard deviation are compared.

### Treatment 1

In treatment 1, the experiment shows how the subjects coordinate on a steady state (see Fig. 1a and d). In treatment 1a, monetary policy is weak (*ϕ*_*π*_ = 1), so that there is no tendency towards the target inflation value, and coordination on a wide range of inflation rates can occur. In treatment 1b, however, under aggressive monetary policy rules (*ϕ*_*π*_ = 1.5), the target inflation plays an important role in the system, so that subjects coordinate on this value when this converging behavior takes place.

**Fig. 3:**
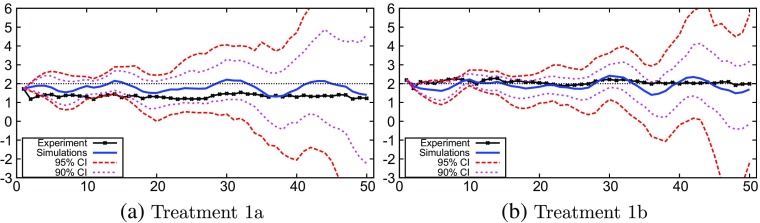
Monte Carlo results: treatment 1

Figure 3 shows the mean and the 90% and 95% confidence intervals of the Monte Carlo simulations together with the experimental data of treatment 1. The mean of the treatment 1b simulation (Fig. 3b) moves around the target inflation, consistent with and close to the experimental data. In contrast, in treatment 1a (Fig. 3a), the mean of the GA-simulation stays below the target inflation consistent with the experimental data.

In both treatments 1a and 1b, the confidence intervals of the GA-simulations slowly increase and become wide towards the end of the experiment, after period 40. These confidence intervals are wider in treatment 1a (Fig. 3a) than they are in treatment 1b (Fig. 3b), showing that drifts of the inflation rate away from the target are more likely to occur under weak monetary policy consistent with the experimental results.

In both treatments 1a and 1b, drifts in inflation and even some oscillatory behavior (in the confidence intervals) are visible, whereas this behavior is absent in the experimental groups (Fig. 1a and d). The standard deviation of the Monte Carlo simulations increases at the end of the simulation period, indicating diverging behavior at least in some GA simulations. The long run drift in inflation and some oscillatory behavior in the GA-simulations is caused by coordination on trend-following behavior and relatively high trend-coefficients *β* (see Fig. 6b below). Furthermore, unstable divergence is still unlikely after 50 periods, as e.g. the 90% confidence interval remains bounded after 50 periods.

### Treatment 2

**Fig. 4:**
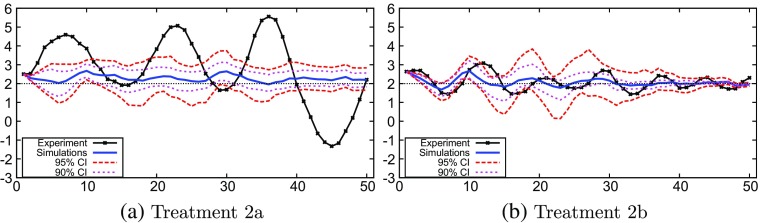
Monte Carlo results: treatment 2

Figure 4 shows the mean and the 90% and 95% confidence intervals of the Monte Carlo simulations together with the experimental data of treatment 2. The mean as well as the confidence intervals of the GA-simulations capture the oscillations in the experimental data of treatment 2. The amplitude of the oscillations in the experimental data in treatment 2a, however, is much larger than in the 50-period ahead GA-simulations and the maxima and minima are outside the 95% confidence intervals (Fig. 4a). Apparently, the long run (50-periods ahead) GA simulations do not capture well the strong coordination on large amplitude oscillations in the experiment, perhaps because the GA-model is too noisy, so that the long run coordination is too weak.

In treatment 2b, the oscillatory pattern of the mean of the GA-simulations matches the experimental results. Although the amplitude of the mean of the GA simulations is somewhat smaller, the experimental data remain within the 95% confidence interval of the GA simulations most of the time (Fig. 4b). Furthermore, as in treatment 1, the difference in monetary policy settings is captured well by the GA simulations. With a weak monetary policy, as in treatment 2a, the magnitude of the confidence intervals of the GA simulations remains more or less constant (with some oscillations). With more aggressive monetary policy, as in treatment 2b, inflation and the output gap are more stable and converge to the target.^9^

### Treatment 3

**Fig. 5:**
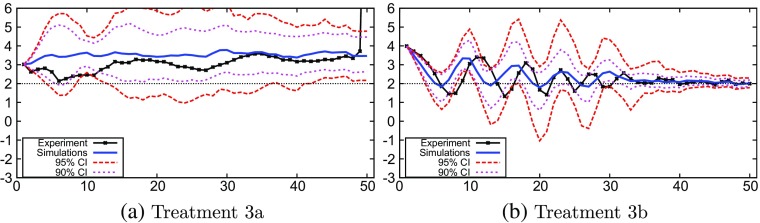
Monte Carlo results: treatment 3

The experimental results of treatment 3a clearly show no attraction to the target inflation (Fig. 5a). This is replicated well by the simulations. The mean of the GA simulations remains almost constant, close to the experimental data. The simulations of treatment 3b replicate the oscillatory behavior that occurs in the experiment (Fig. 4b). Similar to treatment 2b in Fig. 5b, we see that the experimental inflation data remain almost entirely within the 95% confidence interval, while the mean of the Monte Carlo simulations oscillates and closely resembles the experiment. We also see that the dampening of the inflation oscillations under more aggressive monetary policy resembles the dampening in the experiment. Under weak monetary policy in treatment 3a, the confidence intervals of the GA simulations remain wide and above target, while under aggressive monetary policy in treatment 3b the confidence interval initially shows oscillations but stabilizes around the target in the long run after 40 periods.

### Heuristic parameters chosen by the agents

**Fig. 6:**
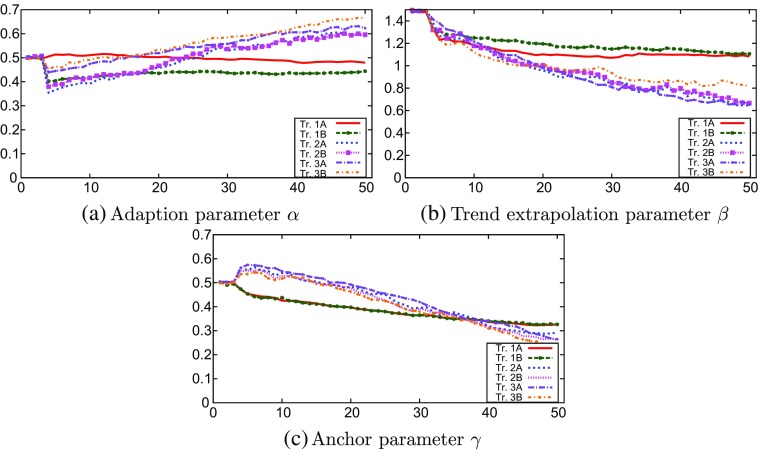
Monte Carlo results: evolution of the chosen heuristic coefficients

Because each treatment yields different dynamics, the coefficients *α*, *β* and *γ* of the forecasting heuristic are optimized differently by the agents. Figure 6a through c compare the time evolution of the mean *α*, *β* and *γ* in the six different treatments; Appendix contains the confidence intervals for all parameters and all treatments over 1000 runs of Monte Carlo simulations. In general, we can say that, in each period in the simulation, the adaptive parameter *α*, becomes more important, while the trend extrapolation *β* and the anchor *γ* become less important. This goes for all treatments. The trend coefficients remain positive and relatively large (≤ 0.65) in all treatments. We do, however, see differences between the different treatments. In particular, for each of the coefficients, treatment 1 differs from treatments 2 and 3. The steeper curves of the optimization parameters in treatments 2 and 3 indicate that the model incentivizes GA agents to update their coefficients faster in these treatments.

**Fig. 7:**
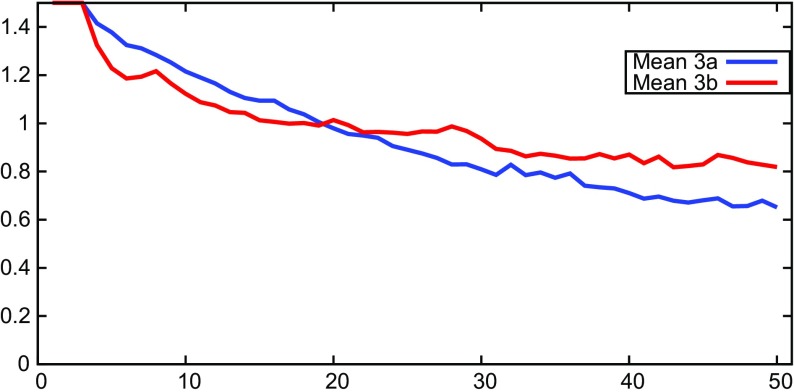
GA model: comparison of the mean trend parameter *β* between treatments 3a and 3b

Figure 7 compares the average trend extrapolating coefficients of treatments 3a and 3b. More aggressive monetary policy (3b) somewhat stabilizes the trend extrapolating coefficients in the short and medium run (the graphs cross at t=19), but not in the long run, where treatment 3b has somewhat stronger trend extrapolation.

### Descriptive statistics

In addition to the confidence intervals of the Monte Carlo simulations, we now compare some simple statistics, the mean and the standard deviation of the experimental data and the Monte Carlo GA simulations. We use the 1000 runs of the Monte Carlo simulations to create a mean and a confidence interval for these three statistics. Table 1 shows that the mean of the experimental inflation rate is close to the mean of the simulated inflation rate. Moreover, in each of the six treatments, the mean experimental inflation rate lies within the confidence interval of the simulations. The model especially captures the difference between the a- and b-session of the treatments. In the b-sessions, the mean is closer to target inflation rate due to a more aggressive monetary policy. This also causes the smaller confidence intervals around or close to the inflation target of 2% in treatments b.

**Table 1:** Mean of the simulated and experimental inflation rate averaged over 50 periods

Treatment	1a	1b	2a	2b	3a	3b
Experimental *μ*	1.3333	2.0719	2.7367	2.0882	3.3728	2.2939
Mean Simulated *μ*	1.7848	1.9042	2.2795	2.0682	3.5199	2.3500
	0.9772	1.3516	1.6637	1.9468	2.1906	2.1426
95% confidence interval	−	−	−	−	−	−
	2.7198	2.3093	2.9664	2.1411	4.8938	2.5496

Table 2 shows how the standard deviation of the experimental inflation rate is replicated by the Monte Carlo simulations. We notice that the mean of the simulated standard deviations differs from the standard deviations in the experiment and that the confidence intervals of the Monte Carlo simulations are large. In treatment 1, the large confidence intervals are especially striking, since the experiments show converging behavior with little variance. In treatments 2 and 3, the mean standard deviation in the simulations is of same order of magnitude as the variance in the experiment. We see that the model overestimates the standard deviation of the inflation rate in the b-sessions, while it underestimates the variance in the a-sessions. Furthermore, the 95% confidence intervals of the b-treatments are almost as large as for the a-treatments, so that this measure does not capture the more stable behavior under aggressive monetary policy in the experimental b-sessions.

**Table 2:** Standard deviation of the simulated and experimental data averaged over 50 periods

Treatment	1a	1b	2a	2b	3a	3b
Experimental *σ*	0.1054	0.1094	1.8136	0.4402	2.3709	0.5978
Mean Simulated *σ*	1.1699	0.9098	0.4541	0.5036	0.7936	0.8242
	0.1287	0.1116	0.1100	0.1512	0.1290	0.3739
95% confidence interval	−	−	−	−	−	−
	5.1134	5.0176	3.0803	3.3741	4.2448	3.6851

Table 2 represents experimental and simulated standard deviations *averaged* over 50 periods. These results may be biased through an initial learning phase of the experiments. To investigate the long run outcomes Table 3 shows the experimental and simulated standard deviations, at the end of the experiment *after* 50 periods. For treatment *b*, with a more aggressive monetary policy rule, the mean after 50 periods is closer to the inflation target of 2% than in treatment a, consistent with experimental data. Furthermore, for treatment *b*, the standard deviation of the simulations is smaller than in treatment *a*. For the GA simulations treatment *b*, with a more aggressive monetary policy rule, is thus more stable than treatment a, consistent with experimental data.

**Table 3:** Mean and standard deviation of the simulated and experimental data **after** *t* = 50 periods

Treatment	1a	1b	2a	2b	3a	3b
Experiment at *t* = 50	1.2191	1.9909	2.2050	2.3067	3.7195 ^10^	1.9941
Mean simulations at *t* = 50	1.4014	1.6919	2.1981	1.9488	3.4677	2.0136
St. dev. simulations at *t* = 50	4.1789	3.2827	0.8036	0.5619	1.3317	0.6992

^10^ In treatment 3a, the experimental inflation at *t* = 50 is very high at 19.6097, due to one extreme forecast. Therefore, we report inflation for *t* = 49.

### One-period ahead forecasts

All GA-simulations presented so far have been long run 50-period ahead simulations. These long run simulations do not use the most recent updated information from the experiment, but only use the initial predictions by the subjects for initialization of the GA-model. The mean and confidence intervals of the 50-period ahead Monte Carlo GA simulations capture most of the experimental results in different treatments fairly well. However, one striking feature of the laboratory experiments *not* captured by the 50-period ahead simulations is the large amplitude fluctuations observed in treatment 2a, which are clearly outside the 95% confidence bands of the 50-period ahead Monte Carlo simulations of the GA-model (see Fig. 4a).

In this subsection, we consider one-period ahead GA-simulations to explain the large amplitude fluctuations in treatment 2a, group 2 (Fig. 1b). The one-period ahead simulations use the most recently observed experimental data, and therefore are based on the same information as available to the subjects in the experiment. Hence, in period *t*, when forecasting inflation in period *t* + 1, one-period ahead GA simulations use inflation up to period *t* − 1, consistent with observable information in the experiment. Instead of long run prediction with a 50-period ahead simulation, the one-period ahead simulation thus “follows” the most recently observed realizations of inflation and other relevant observable macro variables.

**Fig. 8:**
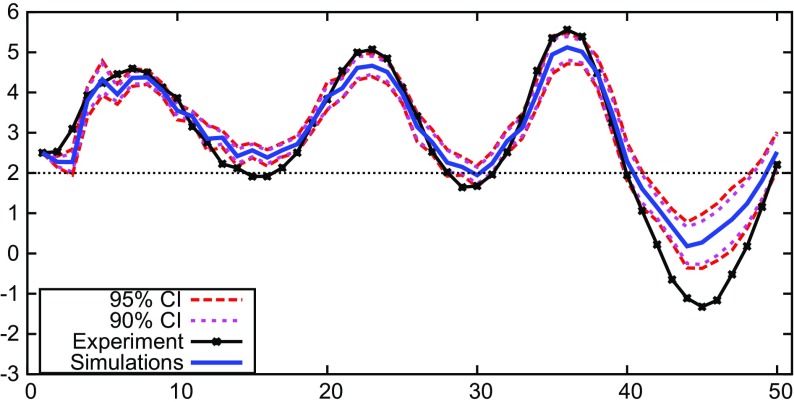
1-period ahead Monte Carlo simulations treatment 2a

Figure 8 shows a one-period ahead forecast simulation of the GA-model of treatment 2a, group 2. The figure shows the mean and the 90% and 95% confidence intervals of 1000 runs of the one-period ahead GA-simulations together with the experimental data. The mean of the one-period ahead GA simulation is fairly close to the experimental data and follows the large fluctuations most of the time. Hence, the one-period ahead GA simulations are able to capture the coordination on large amplitude fluctuations, as observed in the experiment. Only around the local maxima and local minima do the experimental data move outside the 90 − 95% confidence band and the GA one-period ahead simulations do not fully capture the most extreme inflation realizations (e.g., around the global minimum around period 45).

## Conclusions and recommendations

In this paper, we present a genetic algorithm model in which individuals optimize an adaptive, a trend following and an anchor coefficient in a population of general prediction heuristics. With this model, based on Anufriev et al. (2015), we replicate results of a Learning-to-Forecast experiment by Assenza et al. (2013). The experiment investigates how individuals learn to forecast in a New Keynesian macroeconomy with three different treatments, each of them with two different monetary policy settings. The results of this experiment can be classified in three types of aggregate behavior: converging, oscillatory and dampened oscillatory behavior.

We show that a single GA model with a simple set of rules can explain adaptive behavior of human subjects in a predictions feedback environment with varying levels of complexity. As with Arifovic et al. (2012), this paper contributes to understanding learning behavior in a New-Keynesian environment. It furthermore shows that heterogeneous behavior can be explained by an adaptive, anchor and trend extrapolating component. We also contribute to the existing literature that GA can be used to explain heterogeneous behavior in LtF experiments with different types of complexity, but also for macro experiments with two or more aggregate variables, next to more classical ones based on single commodity and financial asset markets.

The 50-period ahead simulations of the GA model are able to replicate these types of behavior from the experimental results. In the first treatment, which typically shows converging behavior, the model clearly captures the difference between the two monetary policy settings. Furthermore, our model clearly replicates the oscillatory and dampened oscillatory behavior from treatment 2b and 3b respectively. In particular, the GA model explains more stable behavior in treatments b, when monetary policy is more aggressive.

The 50-period ahead GA simulations, however, are not able to capture the coordination on large amplitude fluctuations that we see in treatment 2a of the experiment. Apparently, the long run simulations do not explain coordination on large amplitude fluctuations, perhaps because the GA model is too noisy to explain such coordination in the long run. One-period ahead GA simulations, however, using the same recently observed experimental data available to subjects in the experiment, well explain coordination on these large amplitude fluctuations.

If we look at the aggregate outcomes of heuristic parameters *α*, *β* and *γ*, we see a distinction between treatment 1, on the one hand, and treatments 2 and 3, on the other. In both treatments 1a and 1b, there is fast coordination on an equilibrium, after which choices for *α*, *β* and *γ* become almost irrelevant because the inflation and individual predictions of the inflation only slightly fluctuate due to the composite shock term. In treatments 2 and 3, however, we see that *α* increases and *β* and *γ* decrease over time. Nevertheless, the trend coefficient *β* remains positive and relatively large (≥ 0.65), showing that trend-following behavior remains important.

During the procedure of replicating the oscillatory and dampened oscillatory behavior, we find that the model is sensitive to changes in the allowed ranges for the trend extrapolation coefficient. We use a wider range for this parameter than Anufriev et al. (2015). This underlines their finding that different feedback structures lead to different degrees of trend extrapolating behavior. As a result, further work may be necessary to find a generalized version of this model that would capture this aspect of the individual behavior. We also note that the more complicated experimental treatments require some adaptation of the GA model (*c.f*. Anufriev *et al*. 2015, who encountered a similar problem with the two-period ahead non-linear asset pricing economy). This shows that further research should focus on extensions and refinements of this GA model.

## Figures and Tables

**Fig. 9:**
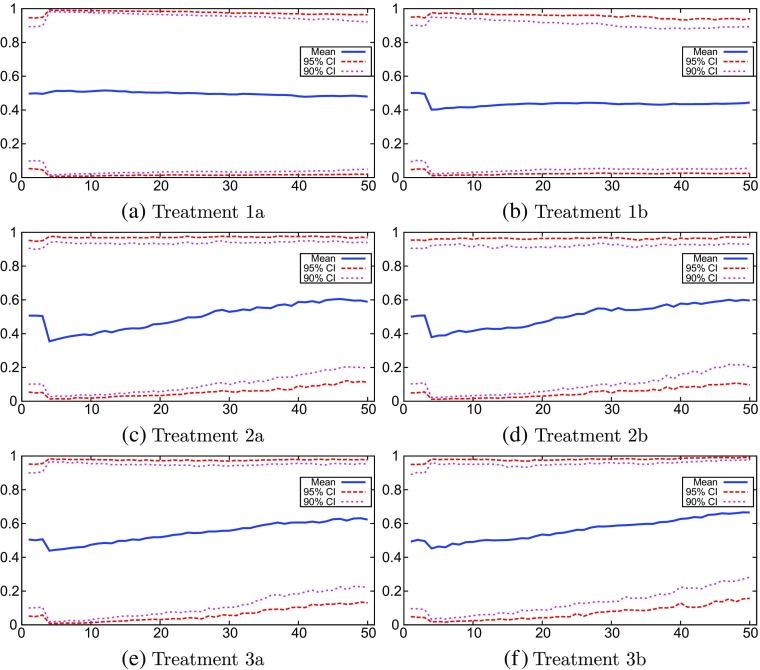
GA model: confidence intervals for the *α* parameter

**Fig. 10:**
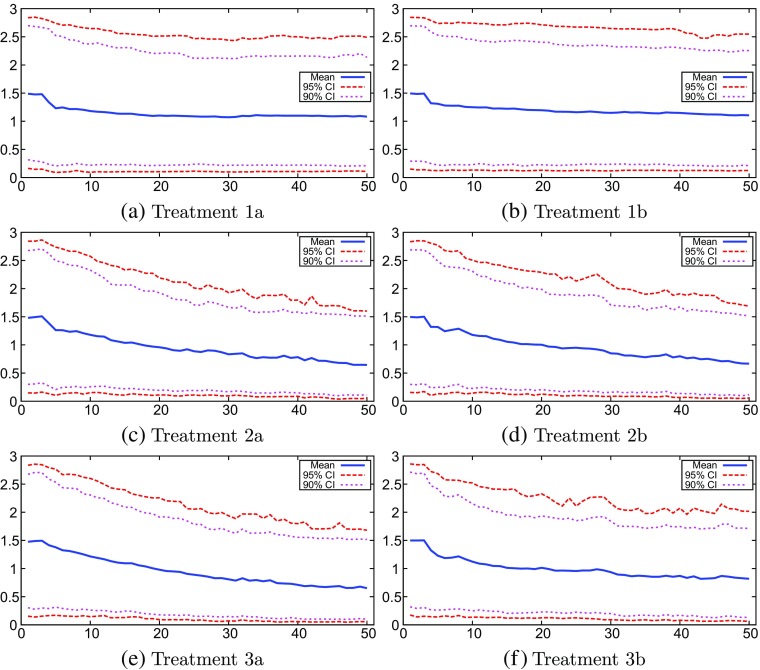
GA model: confidence intervals for the *β* parameter

**Fig. 11:**
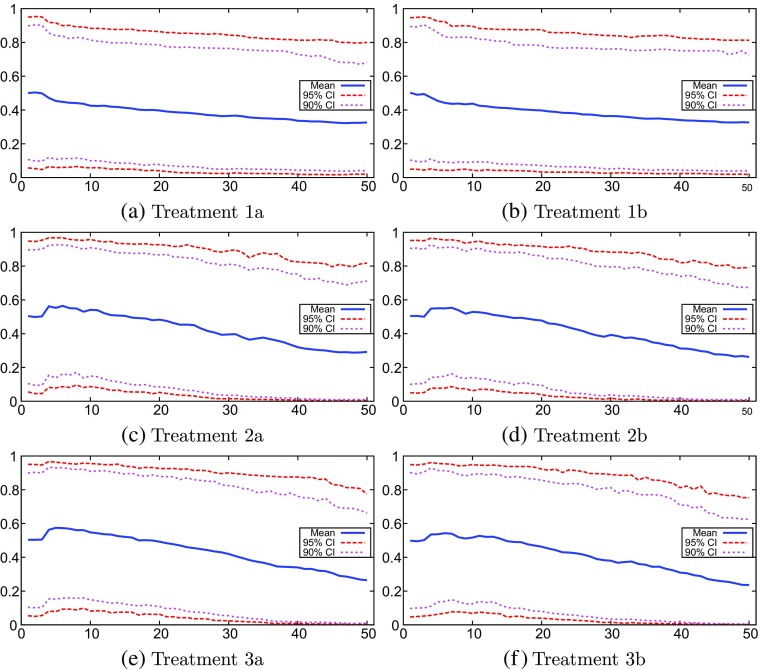
GA model: confidence intervals for the *γ* parameter

## Footnotes

1 Pfajfar and Zakelj (2014) and Kryvtsov and Petersen (2013) also run laboratory experiment within a New Keynesian framework.

2 In the experiment the parameters are fixed at the calibration by Clarida et al. (2000): *ρ* = 0.99, *φ* = 1 and *λ* = 0.3. The inflation target is set to $\overline {\pi }=2$. Coefficient *ϕ*_*π*_ measures the response of nominal interest rate *i*_*t*_ to deviations of the inflation rate *π*_*t*_ from its target $\overline {\pi }$. Error terms *g*_*t*_ and *u*_*t*_ are both normally distributed with *μ* = 0 and *σ* ^2^ = 0.01

3 We take genes to consists of 20 bits. This means that interval [*a*_*n*_ *b*_*n*_] is subdivided into 2^20^ ≈ 10^6^ possible values. In an interval with length 1, say an interval [0 1], the precision in the GA model is then approximately 10^−6^.

4 In this research, the heuristic is a binary representation of multiple coefficients. The crossover is designed such that a subset of these coefficients is interchanged, i.e. the string is not broken up within a coefficient, which means that no new values for coefficients come into existence during the crossover stage. Hence, mutation and crossover have strictly different functions.

5 Subscript *s* is added to this parameter to distinguish the intensity of choice parameter from the trend extrapolation parameter in the heuristics.

6 Recall that in the NK macroeconomic setup, subjects do not predict the present inflation, but the inflation for the next period (i.e. two periods ahead forecasts). Therefore, predictions are needed not only for the first but also for second period. In the second period, subjects observe the inflation in period 1, which they can use in their prediction of the inflation in period 3.

7 The first period of the iteration, however, differs from the following 48 periods, because subjects cannot use trend extrapolation yet. In this period, there is only one observation of the inflation rate and output gap. It is, therefore, assumed that the trend extrapolation coefficient equals zero in this period. After period two, the GA updating continues in full form, where agents can forecast using both past inflation rate and output gap and the past trend or change in the inflation rate and the output gap.

8 We have performed robustness checks with different crossover rates 0.5 and 0.1 instead of 0.9 and mutation rates 0.1 and 0.05 instead of 0.01 and obtained similar results of the GA simulations.

9 Massaro (2012) presents three experimental groups in treatment 2b. We have focused here on group 3 (Figs. 1e and 4b) showing small amplitude fluctuations. Groups 1 and 2 of treatment 2b in Massaro (2012) are in fact more stable and converge to the inflation target, within the 95% confidence intervals of the GA-simulations.
